# Fabrication of 3D-Printed Biodegradable Porous Scaffolds Combining Multi-Material Fused Deposition Modeling and Supercritical CO_2_ Techniques

**DOI:** 10.3390/nano10061080

**Published:** 2020-05-31

**Authors:** Raúl Sanz-Horta, Carlos Elvira, Alberto Gallardo, Helmut Reinecke, Juan Rodríguez-Hernández

**Affiliations:** 1 Institute of Polymer Science and Technology, Spanish National Research Council (ICTP-CSIC), Department of Applied Macromolecular Chemistry, Juan de la Cierva 3, 28006 Madrid, Spain; r.sanzhorta@gmail.com (R.S.-H.); celvira@ictp.csic.es (C.E.); gallardo@ictp.csic.es (A.G.); hreinecke@ictp.csic.es (H.R.); 2 Interdisciplinary Platform for Sustainable Plastics towards a Circular Economy-Spanish National Research Council (SusPlast-CSIC), 28006 Madrid, Spain

**Keywords:** additive manufacturing, biodegradable, biocompatible, supercritical CO_2_, breath figures, microporous materials

## Abstract

The fabrication of porous materials for tissue engineering applications in a straightforward manner is still a current challenge. Herein, by combining the advantages of two conventional methodologies with additive manufacturing, well-defined objects with internal and external porosity were produced. First of all, multi-material fused deposition modeling (FDM) allowed us to prepare structures combining poly (ε-caprolactone) (PCL) and poly (lactic acid) (PLA), thus enabling to finely tune the final mechanical properties of the printed part with modulus and strain at break varying from values observed for pure PCL (modulus 200 MPa, strain at break 1700%) and PLA (modulus 1.2 GPa and strain at break 5–7%). More interestingly, supercritical CO_2_ (SCCO_2_) as well as the breath figures mechanism (BFs) were additionally employed to produce internal (pore diameters 80–300 µm) and external pores (with sizes ranging between 2 and 12 μm) exclusively in those areas where PCL is present. This strategy will offer unique possibilities to fabricate intricate structures combining the advantages of additive manufacturing (AM) in terms of flexibility and versatility and those provided by the SCCO_2_ and BFs to finely tune the formation of porous structures.

## 1. Introduction

The engineering of three-dimensional (3D) porous scaffolds is currently a center of intensive research since these porous materials offer a suitable microenvironment for the incorporation of cells or growth factors for the regeneration of damaged tissues or organs, mimicking in vivo microenvironments where cells interact and behave according to the mechanical and physical cues obtained from the environment. Thus, these scaffolds are commonly used for different biomedical applications [1,2,3] and, in particular, in regenerative medicine [4,5,6,7]. In addition to biocompatibility and biodegradability of the materials there are several major requirements to be accomplished including their precise manufacturing, the minimal toxicity of the degradation products, or a degradation rate that match the recovery rate of the targeted tissue [8,9] and the ability to promote specific events at the cellular level [10]. Finally, they must provide appropriate microenvironments for optimal cell growth and function but also the appropriate mechanical support [9,11,12,13]. For this reason, the desirable 3D scaffolds should be highly porous and with interconnected pore networks to facilitate nutrient and oxygen diffusion and waste removal but also proliferation, and migration for tissue vascularization and formation of new tissues, mimicking, to some extent, the extracellular matrixes (ECM), supporting the growth and regeneration of various cells and human tissues that have been already explored [10,11,14].

An interesting alternative to accomplish these requirements would be the use of rapid and precise fabrication techniques enabling the formation of 3D porous structures using biodegradable materials with different degradation rates [15,16]. In this sense, a wide variety of alternative methodologies to produce porous structures ranging from tens of micrometers up to hundreds of micrometers [17,18] have been already reported and divided into conventional fabrication techniques and additive manufacturing (AM) techniques [14]. Conventional techniques comprise phase separation, gas foaming, salt leaching, or freeze-drying just to mention a few of them, while AM encompasses seven different technologies, the most extended methodologies are [19] fused deposition modeling (FDM) (B), selective laser sintering (SLS), and (C) stereolithography (SLA).

In comparison to AM, conventional fabrication does not allow for a precise control of the internal scaffold architecture or the fabrication of complex architectures. For these conventional methods, the control over the pore size, the pore connectivity, or the final shape of the fabricated part still requires further investigation to be improved. Finally, the above mentioned conventional strategies do not permit the fabrication of a particular 3D shape. In this sense, additive manufacturing technologies (also known as 3D printing techniques) offer unique possibilities to create fully customized scaffolds with high structural complexity and design flexibility [14,16,20]. Few recent examples [21,22] have demonstrated that 3D printing can be combined with other methodologies to produce porous materials with potential interest in tissue engineering (TE) applications, improving the design of the processes that control cell guidance in three-dimensional (3D) materials.

An additional issue is related to the mechanical properties of the final object. Most of the examples reported involve the use of bioprinting, such as Direct Ink Printing (DIW) for the fabrication of scaffolds with interconnected macro and micropores, showing mechanical properties and dimensional stability that are rather limited [23]. For this reason, chemical cross-linking methods are frequently employed to increase mechanical strength [24]. However, chemical cross-linking is often carried out in non-aqueous conditions or requires a previous derivatization of the materials with the appropriate reactive functional groups that may produce cytotoxicity. In spite of all of this, the improvement achieved in terms of mechanical and dimensional stability of scaffolds by cross-linking is often not enough.

Herein, a methodology to prepare multi-material porous (with interconnected internal and external porosity) and biodegradable 3D scaffolds combining (a) conventional methodologies, i.e., gas foaming by supercritical CO_2_/breath figures, with (b) additive manufacturing, i.e., multi-material FDM printing is presented. The main objective is to combine the advantages of each strategy to produce porous 3D-printed objects with micrometer porous sizes (obtained by SCCO_2_) and precisely located porous areas obtained by multi-material FDM printing (in this contribution combining poly (ε-caprolactone) (PCL) and poly (lactic acid) (PLA)). The scaffold will be fabricated by a dual extrusion fused deposition modeling printing (FDM) that enables to straightforwardly fabricate almost any 3D CAD (computer-aided design). By using a precise deposition of PCL and PLA, selectively porous areas will be formed based on our previous reports on the SCCO_2_ foaming of PCL. SCCO_2_ foaming permits the efficient fabrication of microporous scaffolds with pores in the tens of micrometer range in polymers [25,26,27] being CO_2_ removed by simply depressurization of the system and without the use of organic solvents. Finally, today, breath figures (BFs) is an extended methodology to, by water vapor condensation in a moist atmosphere, produce micrometer size porous surfaces [28,29,30,31]. This strategy combining SCCO_2_ treatments and BF, previously described by our research group, will be crucial to obtain interconnection between the pores at the surface and internal pores [32].

Previous examples combining AM and conventional approaches have been limited to the use of one single material so that the mechanical properties could not be modulated and these investigations were exclusively based on the use of PLA. Zhou et al. prepared PLA scaffolds with 100–800 μm macropores by applying fused deposition modeling (FDM) and 1–10 mm micropores generated through gas foaming [21]. Song et al. [22] described the preparation of a PLA based filament with poly (vinyl alcohol) that was employed to fabricate scaffolds by FDM that produced porous scaffolds upon gas foaming and selective solvent etching. To find an ideal single material for an application is rather difficult if not impossible; the scaffold manufacture using combinations of specialist materials can produce more versatile structures [33]. By controlling the percentage and architecture of material components, mechanical properties, cell attachment, and proliferation may be optimized for a given function.

In addition to the limitations of using a single material, none of the above mentioned examples take into account the ’so-called’ skin layer formed when using SCCO_2_ foaming processes. This layer is a major limitation in the use of these scaffolds since there is no connection between the environment and the pores so that cells cannot migrate towards the implant. In this research, by creating a surface porosity using the breath figures approach the skin layer will be removed, obtaining porous interconnected polymer samples.

In this manuscript, we report for the first time a strategy that combines additive manufacturing with two complementary methods (i.e., SCCO_2_ and breath figures) to prepare a 3D-printed object with internal and external interconnected porosity. Moreover, instead of using a single material we describe the preparation of multi-material scaffolds that, as will be described, allowed us to precisely define the porous and solid areas.

## 2. Experimental Section

### 2.1. Materials

Facilan™PCL100 Poly (ε-caprolactone) filament of 1.75 mm diameter was purchased from 3d4makers (Haarlem, The Netherlands) and natural poly (lactic acid) 1.75 mm diameter filament was purchased from Filament2print (Nigrán, Portugal). Liquid carbon dioxide was supplied by Carburos Metálicos (Cornella de Llobregat, Spain) with a 99.99% purity, and chloroform (CHCl_3_) was supplied by Sigma Aldrich.

### 2.2. Methods

#### 2.2.1. Fabrication of Multi-material 3D Parts Combining PCL and PLA

Parts were designed on the Autodesk Inventor 2018 CAD software obtaining a *.stl* file that was later processed on IdeaMaker version 3.3.0 to obtain a *.Gcode* file in which printing parameters were defined for a single or multi-material 3D printing process as is shown in Table 1. Parts were printed on a Raise3D Pro 2 printer and a 0.4 mm diameter nozzle was used for the printing process.

#### 2.2.2. Supercritical CO_2_

Printed parts were placed in a Thar R100W 104 mL SCCO_2_ reactor where CO_2_ was pumped from a CO_2_ tank to a high pressure pump pre-cooled using a cryostat at 4 °C. Desired pressure and temperature were maintained by the automatic back pressure regulator (ABPR). The system remained at these conditions for a processing time of 90 min using a CO_2_ flow of 5 g/min for all the cases, and then depressurization took place until atmospheric pressure. Temperature was varied from 31 to 35 °C and pressure was maintained at 200 bar due to the previous optimization of the parameters.

#### 2.2.3. Breath Figures

Samples were immersed in CHCl_3_ during different times (1–15 s) under saturated relative humidity (100%) conditions at room temperature in a closed chamber.

Samples treated by SCCO_2_ were fractured to analyze their cross-section by scanning electron microscopy SEM (XL30ESEM Philips, North Billerica, MA, USA) at an acceleration voltage of 25 kV. Images taken by SEM were analyzed by ImageJ (NIH, USA), a Java-based image processing program, to measure pore size, pore morphology, and foamed region. Breath figures-treated samples were also analyzed by SEM to measure pore sizes on the surface of treated parts. Average pore sizes were determined by measuring the diameter of 30 pores of the image and obtaining their average and standard deviation.

#### 2.2.4. Mechanical Characterization: Uniaxial Tensile Test

The mechanical properties were analyzed in an electromechanical Instron 3366 (DA, Germany) system which performed uniaxial tensile tests. The specimens printed on PLA, PCL, or a combination of both were designed by following ISO37:2011 prescript (type 3 dimensions). Samples made of just one material (PLA or PCL) were printed with a varying layer height (0.1–0.25 mm) and position of the samples when printed (horizontal or vertical). When multi-material samples were prepared, a number of shells printed in PCL were varied and a 99% infill of PLA was maintained in order to observe differences in its mechanical properties.

#### 2.2.5. Computerized Tomography (Micro-CT)

The interconnectivity of the pores as well as the internal structure of the samples was studied by micro X-ray computed tomography with the unit CT-SCAN-XT-H-160 (NIKON, Tokio, Japan). A PCL-foamed screw and a PLA/PCL-foamed screw were analyzed to obtain a volumetric 3D reconstruction of the whole sample with a range of colors that defines the interconnectivity between pores.

### 2.3. Results and Discussion

**Figure 1:**
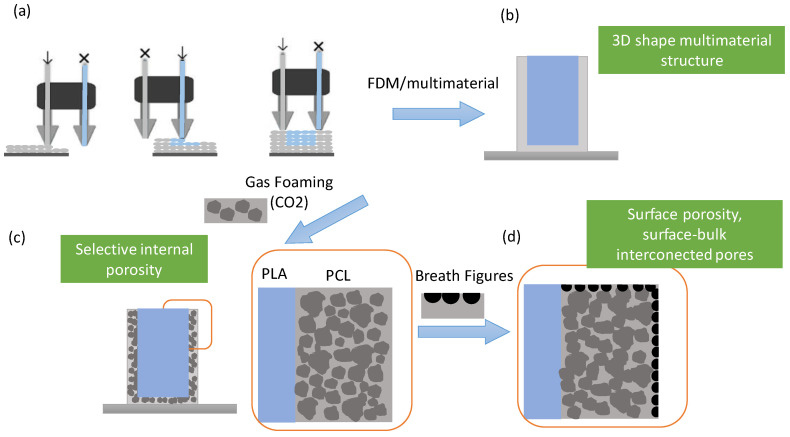
Strategy employed to fabricate 3D multi-material porous scaffolds with internal and external porosity. (**a**,**b**) Multi-material 3D printing of poly (lactic acid) (PLA)/poly (ε-caprolactone) (PCL) parts, (**b**,**c**) foaming of the parts using supercritical CO_2_ (SCCO_2_), (**c**,**d**) surface porosity formed using the breath figures approach.

The strategy proposed to prepare multi-material 3D porous scaffolds involves three consecutive steps (Figure 1). First, the fabrication of the 3D-printed parts that will be carried out by multi-material FDM 3D printing. In addition to the combination of PLA and PCL, parts exclusively printed with each material will be fabricated. The second step involves the foaming of the 3D-printed structures by using the SCCO_2_ technique. Finally, in order to create pores in the ’so-called’ skin layer (typically observed as the result of the SCCO_2_ process), a surface treatment, i.e., the breath figures approach was employed to produce surface porosity interconnected with the internal porosity.

**Figure 2:**
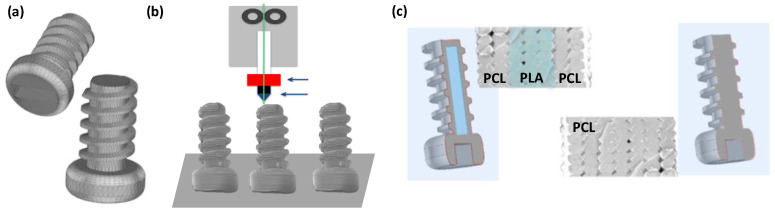
Strategy followed for the preparation of 3D-printed structures with internal and external porosity: (**a**) design: CAD software, (**b**) fabrication: additive manufacturing (AM), (**c**) structure and materials employed for the two different prototypes.

For the fabrication of the 3D-printed parts, the design of the 3D structures was achieved by using a computer-aided design (CAD) software (Figure 2). To illustrate the feasibility of this strategy, a screw structure was employed as a model. The models were printed by fused deposition modeling (FDM) either using exclusively PCL or combining an internal PLA structure and an external PCL structure. The rationale behind this selection of degradable materials relies on different aspects. First, the biocompatibility of both materials (FDA approved) enable us to test these parts for their use in biomedical purposes [34]. Secondly, PCL is non-toxic and tissue compatible, and hence widely used as absorbable sutures, as scaffolds in regenerative therapy, and in drug delivery applications. PCL exhibits a longer degradation time (2–3 years) in comparison to PLA and is degraded by microorganisms or by hydrolysis of its aliphatic ester linkage under physiological conditions [35,36]. Third, PCL has been previously employed in our group and successfully foamed by using the SCCO_2_ approach and surface treated with the breath figures methodology [31]. Finally, PCL and PLA present, in terms of mechanical properties, very different behaviors. At physiological temperature, the semi-crystalline PCL attains a rubbery state resulting in its high toughness [37] and high elasticity. PLA, in contrast, is rather rigid and breaks upon deformations of 8–10% [35]. Their combination will enable, as will be depicted later, the fabrication of objects with mechanical properties that can be gradually varied.

**Figure 3:**
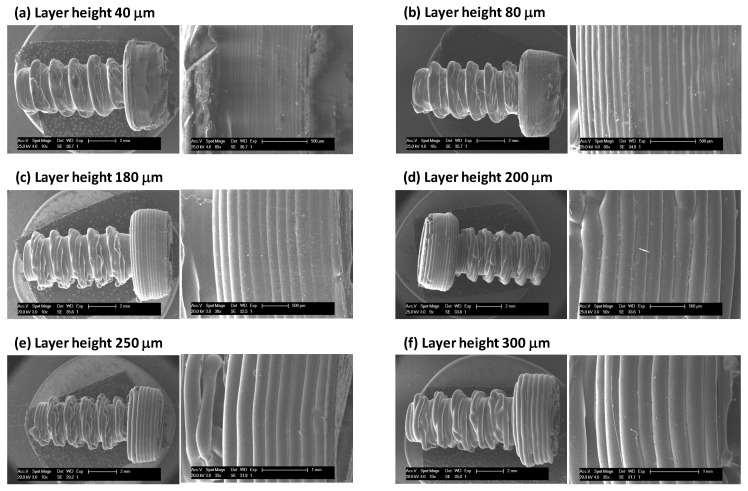
SEM images of the fused deposition modeling (FDM) fabricated of PCL screws with a variable layer height ranging from (**a**) 40 μm, (**b**) 80 μm, (**c**) 180 μm, (**d**) 200 μm, (**e**) 250 μm and (**f**) 300 μm.

Temperatures, printing speed, or layer height are few of the aspects that were first optimized to prepare high quality parts (see experimental section). The first series of experiments were conducted to fabricate the 3D-printed screws. For instance, PCL and PCL/PLA screws were prepared with a variable layer height ranging from 40 to 300 μm. In Figure 3, the SEM images for the different screws fabricated at 115 °C and a speed of 5 mm/s are shown. It is worth mentioning that within the range explored, the layer thickness has been perfectly reproduced while the quality of the thread appears to be optimal in the range of 80 to 200 μm.

Provided the optimized printing parameters (including temperature, printing speed, or flow), and selected the layer height to be 200 μm for all the rest of the experiments, 3D-printed parts with either PCL or with a combination of PLA/PCL were fabricated for the foaming step using supercritical CO_2_.

For the gas foaming step, the 3D-printed parts were initially saturated with CO_2_ at a certain temperature (31 °C) and pressure (200 bar) (see experimental section). As reported by Zhou et al. [38], at the temperatures and pressures employed, CO_2_ diffuses into the polymer matrix until a two-phase CO_2_/polymer solution equilibrium is reached. By either increasing the temperature or reducing the pressure, the equilibrium can be a thermodynamically unstable state, so that the absorbed CO_2_ nucleates, forms bubbles, and finally leads to micropores within the polymer [39].

According to previous studies in which the gas foaming theory has been described [40], the selected processing parameters are the critical aspect to finely tune and manipulate the pore characteristics (including morphology and pore size). In this sense, foaming temperature, saturation pressure, or the pressure drop rate can be modified, which enables a precise control over the final structure. In the particular case described herein, those parameters that enable the selective formation of micropores in the PCL parts will be explored. It is worth mentioning that PLA-printed parts were tested in previous experiments and, under the conditions employed for the foaming of PCL (temperature 31 °C, pressure 200 bar), the parts remain unaltered. According to other reports, PLA requires temperatures from 100 to 140 °C to foam [41].

**Figure 4:**
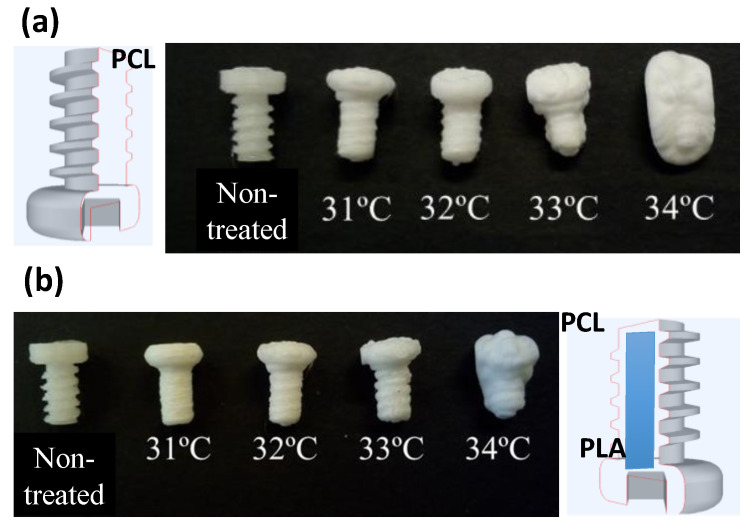
Optical photographs of the 3D-printed parts upon treatment in SCCO_2_ at different temperatures. (**a**) Screws fabricated using PCL, (**b**) screws fabricated using a combination of PCL and PLA (protocol: 200 bar, 5 min).

As has been mentioned, one of the critical aspects is the temperature of the reactor. As shown in Figure 4, the treatment of the screws fabricated in PCL could only be carried out at temperatures around 31 °C. At temperatures above 31 °C, PCL is not only completely swollen by the CO_2_, but PCL is significantly deformed losing partially the 3D-printed structure. At temperatures above 34 °C, the shape of the printed part is completely lost. However, in the case of screws with internal PLA layers and external PCL shells, the temperature of the SCCO_2_ treatment could be increased up to 32–33 °C without affecting the printed structure. This clearly indicates that the PLA improves the resistance of the object during the foaming process.

**Figure 5:**
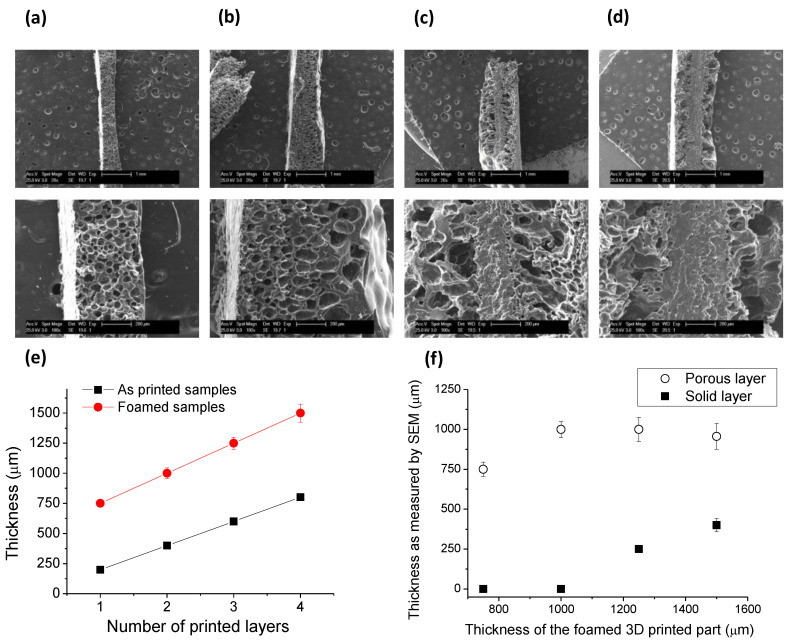
Cross-section SEM images of 3D-printed samples with a variable thickness of (**a**) 200 µm, (**b**) 400 µm, (**c**) 600 µm, and (**d**) 800 µm. (**e**) Representation of the thickness of the printed part (as printed) versus the foamed part. (**f**) Representation of the porous layer and the solid inner layer as a function of the thickness of the printed part foamed. (SCCO_2_ treatment conditions: 200 bar and 31 °C).

Taking into account the limitation in terms of temperature that can be employed in the SCCO_2_ process, i.e., 32 °C in the case of pure PCL-printed parts and 33 °C in dual PCL/PLA parts, the extent of the treatment (the depth of the microporous layer) produced during the foaming process using these conditions was analyzed. For this purpose, planar samples with variable thicknesses were prepared printing from 1 up to 4 layers of PCL with 200 µm for each layer, i.e., 200–800 μm parts were treated using the same experimental conditions. In order to analyze the extent of the foaming process, cross-sectional profiles of the foamed parts were analyzed by SEM. In Figure 5a–d, the cross-sections of the 3D-printed parts with a variable number of layers are presented. In this series of images, it can be clearly seen that for the thinnest parts printed (1 and 2 layers) a complete and homogeneous foaming is obtained. However, those foamed samples formed by more of three layers presented a solid area in the center of the object and a foamed outer part. This result clearly indicates that the CO_2_ is not able, in the conditions employed, to diffuse into the entire part. Moreover, the thickness of the solid inner layer observed gradually increased with the total thickness of the printed part. The measurements of total thickness before and after SCCO_2_ treatment and the thickness of the solid and the foamed areas are illustrated in Figure 5e,f. As expected, the total thickness of the sample is in all cases larger for those samples exposed to the SCCO_2_ treatment. However, as depicted in Figure 5f, the average thickness of the foamed part grows up to ~1 mm in films prepared by foaming 200 and 400 µm thick printed parts but remains constant for thicker films. This clearly indicates that the CO_2_ diffusion is limited to the first 500 µm in the experimental conditions that, as has been explained before, are restricted in temperatures of up to 32 °C.

**Figure 6:**
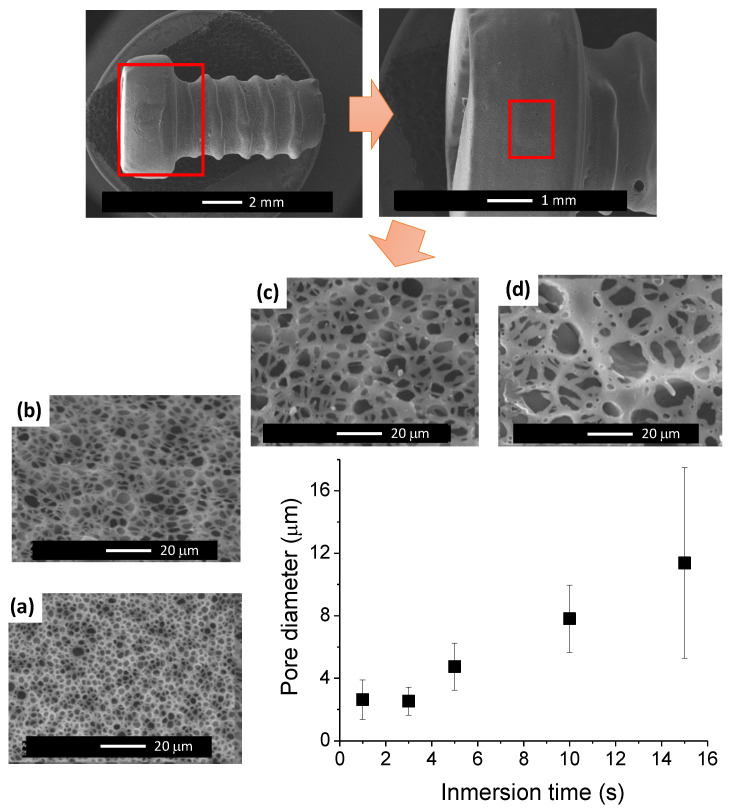
SEM images of the screw surface upon breath figures treatment immersing the sample in chloroform for (**a**) 3 s, (**b**) 5 s, (**c**) 10 s, and (**d**) 15 s.

As has been largely depicted in the recent literature, one of the major limitations of SCCO_2_ treatment is related to the ’so-called’ skin layer that is associated to the rapid diffusion of the embedded fluid out of the sample edges which results in the formation of this dense nonporous skin layer. While the thickness of this layer can be decreased to a certain extent, for instance, by an increase in pressure [42], the complete removal of the skin layer still remains a major issue. In order to overcome this limitation, the breath figures approach was employed to remove this skin layer and enable the preparation of porous scaffolds with continuous internal–external porosity [32]. The breath figures approach has been largely employed to produce microporous surfaces by evaporation of polymer solutions using a volatile solvent. Experimentally, this strategy requires the dissolution of the polymer and evaporation in a moist atmosphere. As a result, during the evaporation, the water vapor is able to condense and form water droplets at the polymer solution/air interface. Finally, these water droplets evaporate and leave microporous cavities at the surface. While this strategy has usually been carried out using polymeric solutions, an alternative to this involves the preparation of microporous surfaces by dip-coating. In this case, a solid polymeric part is immersed for a short period of time (seconds) in the appropriate solvent, removed from the solvent, and allowed to dry in a closed vessel chamber. The relative humidity or the immersion time are among the parameters that play a critical role of the micropore formation at the surface of the polymer part. In this study, the relative humidity was maintained constant at values between 95–100% r.h. to assure the presence of a large amount of water vapor that can be condensed at the surface. While the relative humidity was held constant, the immersion time was varied between 1 and 15 s. In Figure 6, the SEM images of 3D-printed and foamed screws that were treated using the breath figures approach varying in immersion time are depicted. As can be observed in the images in Figure 6a–d, the pore size clearly increased by increasing the immersion time. In fact, a larger immersion time associated with a deeper swelling of the polymer surface, accumulating more solvent, and thus enabling the evaporation process to occur during a longer period of time. As a result, the water vapor can be condensed during longer periods of time, forming larger water droplets, and finally, upon evaporation of the water droplet, larger pores. In the graph presented in Figure 6, a gradual variation of the micropore diameter between 3 μm and 12 μm is evidenced. Equally, the heterogeneity in terms of size gradually increased producing a rather disperse distribution of pores for the largest immersion time employed (15 s).

**Figure 7:**
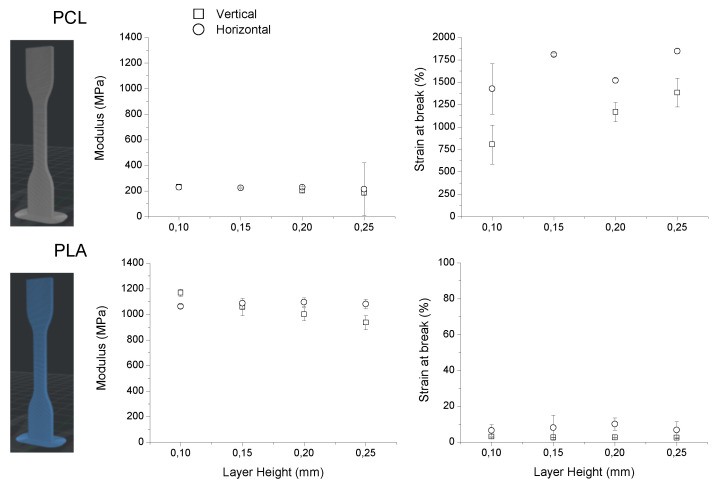

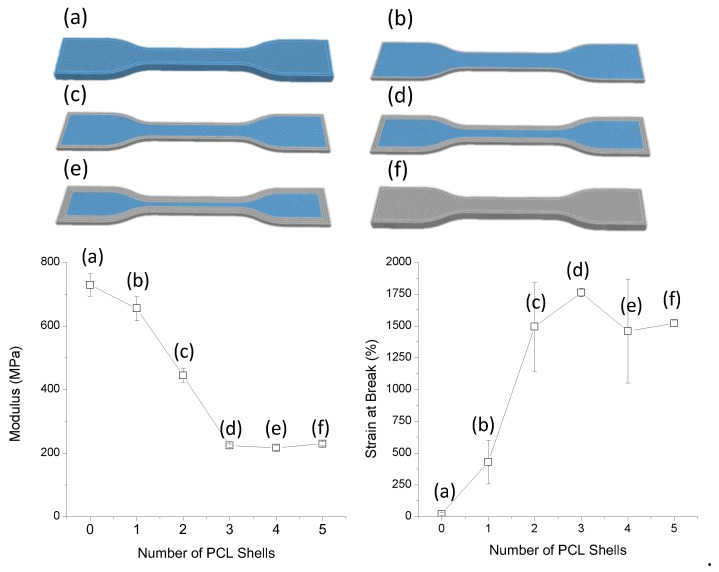
Top: modulus (MPa) and strain at break (%) for PCL. Middle: modulus (MPa) and strain at break of PLA-printed parts. (**a**–**f**) Illustrative pictures of the horizontally printed specimens with a number of external PLA layers gradually substituted by PCL. Bottom left: modulus (MPa) of the specimens versus de number of PCL shells. Bottom right: strain at break (%). Layer height: 200 μm.

In addition to the evaluation of the internal/external pore formation using the combination of SCCO_2_ and breath figures, the mechanical properties of standardized specimens printed using PLA, PCL, and a mixture of both was investigated. First of all, the mechanical properties (modulus and strain at break) of horizontally and vertically printed specimens for both PLA and PCL were analyzed using different layer thickness (from 0.1 to 0.25 mm) (top and middle of Figure 7). Interestingly, these two materials present completely different properties. PCL-printed parts present a very low modulus of around 200 MPa, and this range of values appears to be similar for both printing orientations. In contrast to this low modulus, the strain at break of PCL-printed parts is rather high with values of up to 1700%. In this case, slight differences were observed between the two different printed orientations. It appears that horizontally printed specimens present a higher strain at break in PCL samples. In the case of PLA, slight differences were observed depending on the printing direction. While the modulus of the specimens printed horizontally remain constant for the layer thicknesses evaluated, the vertically printed parts presented moduli that decreased with the layer thickness from 1200 MPa down to 900 MPa. However, independently of the slight differences observed, the moduli measured are around 5 times higher than those measured for PCL. Concerning the strain at break, the PLA specimens present values of less than 10%.

In conclusion, the mechanical properties of the printed specimens of each material present properties of moduli and strain at break that are somehow complementary. PCL parts present a low modulus and a high strain at break, and PLA has a comparatively high modulus with a very low strain at break. Thus, taking advantage of a multi-material FDM printer. The next series of experiments focused on the preparation of specimens with a combination of the two materials selected to modulate (within the mechanical properties of the materials employed) the final mechanical properties of the printed object.

**Figure 8:**
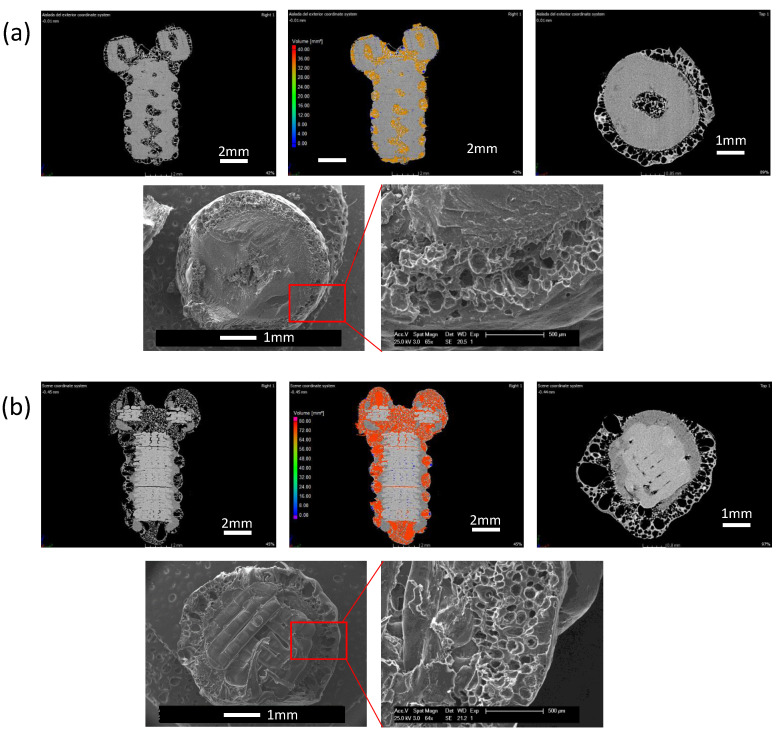
Micro-Computerized Tomography (CT) (above) and SEM (below) cross-sections of illustrative examples in which the samples present a solid internal structure and a porous area limited to the surface layers. (**a**) Screw fabricated in PCL with porous surface. (**b**) Screw with an internal PLA solid structure and a porous PCL external layers.

As depicted in the bottom part of Figure 7, the specimens were printed horizontally in this case, and the number of shells (external layers) were gradually substituted from PLA to PCL (bottom of Figure 7a–f). Therefore, multi-material specimens with 1 to 4 shells of PCL and internal PLA structure were fabricated with the objective to modulate the mechanical properties of the final structure. According to the results of modulus and strain at break represented in Figure 8, a gradual decrease of the modulus is observed by introducing up to 3 shells of PCL and is stabilized at values close to those found for pure PCL in multi-material specimens prepared with 3 or more PCL shells. The same behavior was observed for the strain at break measurements. In this case, the values gradually increased in those samples with increasing number of PCL shells. Equally, in this case, the values are stabilized around 1500–1700% in those specimens prepared using 3 or more PCL shells.

Finally, micro-CT in combination with SEM was employed to accurately characterize the porous areas in the 3D-printed screws. In Figure 8, the micro-CT Z-Y and X-Y cross-sectional profiles as well as a SEM image of the X-Y section of two 3D-printed screws are depicted. In Figure 8a, a PCL screw foamed at 31 °C was analyzed. It can be observed that only the superficial region of the samples foamed as CO_2_ is not able to dissolve completely in the whole polymeric sample, using those parameters (temperature 31 °C and pressure 200 bar). However, it can be observed from Figure 8a that the thread of the screw remained intact after the foaming process. This interesting effect can be associated to the same reason why a non-porous skin layer forms on the surface of SCCO_2_-treated samples: the geometrical shape of the thread has more surface/volume ratio than the non-threaded region of the screw enabling the rapid diffusion of CO_2_ out of that region of the sample. Therefore, this event demonstrates that porosity can be modulated by both varying the SCCO_2_ parameters and also by changing the shape of the samples.

In Figure 8b, the 3D-printed PCL/PLA screw was foamed at 32 °C and, as can be identified, the PCL layers were entirely porous. On the contrary, the PLA structure can be easily identified in the center of the cross-section and remains unaltered without any porosity. On the right side of Figure 8a,b, the pores colored in red indicate that they are interconnected between each other promoting a porous network along the sample, and blue pores are the ones that are isolated from the rest of the porous network. This high interconnection between pores offers a good candidate for future cell cultures.

## 3. Conclusions

The fabrication of 3D-printed porous materials prepared combining additive manufacturing (fused deposition modeling), a foaming methodology (using supercritical CO_2_ as foaming agent), and the breath figures approach (to remove the ’so-called’ skin layer formed in the SCCO_2_ foaming process) has been described.

Multi-material fused deposition modeling (FDM) screws—employed as models for this study—combining poly (ε-caprolactone) (PCL) and poly (lactic acid) (PLA) were fabricated. The use of 3D-printed multi-materials permitted to construct objects with solid and porous areas based on the CAD design, taking into account that the foaming step can be selectively induced in the PCL areas. Moreover, the materials selected clearly illustrate the wide myriad of possibilities to finely tune the mechanical properties of the 3D-printed part depending both on the structural design but also on the proportion of each material and their particular position in the object. We evidenced that, depending on the part composition with a variable amount of PLA/PCL, the modulus can be varied between 200 MPa and 1.2 GPa for 3D-printed parts formed exclusively by PCL to parts comprising only PLA. Equally, the strain at breath significantly decreases from 1700% to 5–7% when the amount of PLA in the printed part increases.

Finally, supercritical CO_2_ (SCCO_2_) as well as the breath figures technique (BFs) were additionally employed to produce internal and external pores exclusively in those areas where PCL is present. Internal pores with sizes ranging between 80 and 300 µm were obtained and interconnected with external pores with sizes ranging between 2 and 12 µm depending on the immersion time.

This strategy will offer unique possibilities to fabricate intricate structures combining the advantages of AM in terms of flexibility and versatility and those provided by the SCCO_2_ and BFs to finely tune the formation of porous structures.

## Figures and Tables

**Table 1:** Printing parameters for the prepared materials.

Material	Printing Temperature (°C)	Printing Speed (mm/s)	Fan Speed (%)	Heated Bed Temperature (°C)	Layer Height (mm)
**PCL**	115	5	100	30	0.04–0.3
**PLA**	215	5–20	100	30	0.04–0.2
**PCL/PLA**	115/215	5–10	100	30	0.2
